# Establishing the Feasibility of Group Metacognitive Therapy for Anxiety and Depression in Cardiac Rehabilitation: A Single-Blind Randomized Pilot Study

**DOI:** 10.3389/fpsyt.2020.00582

**Published:** 2020-06-30

**Authors:** Adrian Wells, David Reeves, Calvin Heal, Peter Fisher, Linda Davies, Anthony Heagerty, Patrick Doherty, Lora Capobianco

**Affiliations:** ^1^ Faculty of Biology, Medicine and Health, School of Psychological Sciences, The University of Manchester, Manchester, United Kingdom; ^2^ Research and Innovation, Greater Manchester Mental Health NHS Foundation Trust, Manchester, United Kingdom; ^3^ NIHR School for Primary Care Research, Manchester Academic Health Science Centre, The University of Manchester, Manchester, United Kingdom; ^4^ Institute of Psychology, Health and Society, University of Liverpool, Liverpool, United Kingdom; ^5^ Liverpool Clinical Health, The Royal Liverpool and Broadgreen University Hospital NHS Trust, Liverpool, United Kingdom; ^6^ Centre for Health Economics, Division of Population Health, Health Services Research and Primary Care, Faculty of Biology Medicine and Health, School of Health Sciences, The University of Manchester, Manchester, United Kingdom; ^7^ Core Technology Facility, The University of Manchester School of Medical Sciences, Manchester, United Kingdom; ^8^ Manchester University NHS Foundation Trust, Manchester Royal Infirmary, Manchester, United Kingdom; ^9^ Department of Health Sciences, University of York, York, United Kingdom

**Keywords:** cardiac rehabilitation, anxiety, depression, mental health, metacognitive therapy

## Abstract

**Background:**

Anxiety and depression are common in cardiac rehabilitation (CR) patients. However, CR programs which incorporate psychological techniques achieve modest reductions in emotional distress. More efficacious interventions that can be easily integrated within services are required. A promising alternative to current psychological interventions is metacognitive therapy (MCT). The aim was to evaluate the acceptability and feasibility of delivering Group-MCT to CR patients experiencing symptoms of anxiety and depression.

**Method and Results:**

Fifty-two CR patients with elevated anxiety and/or depression were recruited to a single-blind randomized feasibility trial across three UK National Health Service Trusts and randomized to usual CR or usual CR plus six weekly sessions of group-MCT. Acceptability and feasibility of adding group-MCT to CR was based on recruitment rates, withdrawal, and drop-out by the primary end-point of 4 months; number of MCT and CR sessions attended; completion of follow-up questionnaires; and ability of the outcome measures to discriminate between patients. The study was also used to re-estimate the required sample size for a full-scale trial. We also examined the extent by which non-specialists adhered to the Group-MCT protocol. Group-MCT was found to be feasible and acceptable for CR patients with anxiety and depression. Recruitment and retention of participants was high, and attendance rates at CR were similar for both groups.

**Conclusion:**

The results suggest the addition of MCT to CR did not have a negative impact on retention and support a full-scale trial of Group-MCT for cardiac patients.

## Introduction

Heart and circulatory disease is associated with approximately 170,000 deaths each year in the UK (1). However, survival rates are improving, and approximately 7.4 million people are living with heart and circulatory disease in the UK (1). Rehabilitation following a cardiac event is vital for improving health outcomes, quality of life, and survival. The UK Department of Health, National Institute for Health and Care Excellence (NICE) guidelines (CG172, CG94, and NG106), and the British Association for Cardiovascular Prevention and Rehabilitation (BACPR) recommend that cardiac rehabilitation (CR) programs are offered to all eligible patients (2–4).

Modern CR programs are comprised of exercise training, psychosocial support, risk factor management, and education underpinned by a behavior change approach (2). CR is cost-effective (5, 6), reduces mortality and morbidity (5, 7), and increases quality of life (5, 8). However, improvement of anxiety and depression in cardiac rehabilitation is limited. Anxiety and depression are highly prevalent among CR patients, with 27.6% of CR patients experiencing clinically significant anxiety and 19% experiencing clinically significant depression (9). Furthermore, elevated anxiety and depression have been associated with decreased medication adherence, decreased CR attendance (10), decreased quality of life, increased risk of reoccurrence of cardiac related events, and a higher rate of mortality, leading to greater service use and increased NHS costs (11, 12).

Combined with the low rates of provision of psychological treatment, those treatments that have been evaluated appear to have limited efficacy. Current interventions for anxiety and depression for patients with a physical illness often focus on cognitive behavioral therapy (CBT). A recent Cochrane systematic review and meta-analysis evaluated psychological interventions in coronary heart disease in comparison to treatment as usual, and included 35 studies. Common components of psychological interventions included relaxation techniques, self-awareness and self-monitoring, emotional support/client-led discussion, and cognitive challenging/cognitive restructuring. At post-treatment interventions were associated with a between group effect size of 0.27 (Cohen's d) for symptoms of depression, with similar results for symptoms of anxiety (Cohen's d = 0.24) (13). This is in line with previous studies that highlight the limited efficacy of psychological interventions in cardiac patients (14, 15). It is evident that more effective interventions that can be easily integrated within services are required.

Metacognitive therapy (MCT) (16), is a transdiagnostic therapy found to be efficacious in mental health settings [see (17)]. MCT might be well suited to the psychological needs of cardiac patients because it does not depend on challenging the validity of negative thoughts and beliefs which can be realistic in people suffering from chronic and potentially life-threatening conditions. MCT is based on the self-regulatory executive function model (S-REF model) (18, 19) of psychological disorders. In the S-REF model, anxiety, depression, and adjustment difficulties are maintained by the activation of a maladaptive thinking style called the cognitive attentional syndrome (CAS). The CAS is characterized by worrying, rumination, inflexible attention to threat, and maladaptive coping strategies. The CAS is driven by underlying metacognitive beliefs, which can be differentiated into positive and negative subtypes. Positive metacognitive beliefs concern the usefulness of worry (e.g. “worrying helps to detect problems before it is too late”) while negative metacognitive beliefs concern uncontrollability (e.g. “I cannot stop worrying about the future”) and dangerousness of worry (e.g. “worrying will cause a heart attack”).

Normann and Morina (17) conducted a systematic review and meta-analysis of the efficacy of metacognitive therapy for anxiety and depression and found that MCT demonstrated a large between group effect size at post treatment (Hedges' g = 2.06) when compared to a waitlist control condition. When MCT was compared to cognitive and behavioral interventions MCT also demonstrated large effect sizes favoring MCT at post treatment (Hedges' g = 0.69). MCT has also shown promising results when delivered using a group format in mental health settings. To date there have been nine studies evaluating Group-MCT across various disorders including obsessive compulsive disorder (20, 21), generalized anxiety disorder (22–24), depression (25, 26), and in mixed disorder groups (27, 28). No studies to date have compared Group-MCT with individual MCT, but a preliminary comparison of Group-MCT with group mindfulness based stress reduction in mixed anxiety and depression groups found that both were feasible and acceptable, with large within-subject effects (27). In physical health conditions MCT has primarily been evaluated in cancer, with promising pilot results (29–31).

While positive outcomes for MCT within mental health settings are rapidly gaining support, accelerated research efforts are needed in the application to treating psychological distress symptoms in physical health. As such the aims of the current study were to evaluate the acceptability and feasibility of delivering group metacognitive therapy within cardiac rehabilitation services, the present study was intended to serve as a feasibility for a subsequent definitive trial [trial protocol: (32)]. Our research questions were: (1) What are the recruitment and retention rates? (2) Do therapists adhere to the treatment protocol? (3) What proportion of the treatment (i.e. CR and Group-MCT) is completed by patients? (4) What is the rate of return of measures and the variability in scores for primary and secondary outcomes? (5) Are there any adverse events or serious adverse events in the intervention group? (6) What is the sample size required for a full-scale trial?

## Methods

### Design

The PATHWAY Group-MCT pilot feasibility study is a multicenter randomized controlled trial with 4- and 12-month follow-up comparing Group-MCT plus usual cardiac rehabilitation (intervention) versus usual cardiac rehabilitation (control). The study served as an internal pilot to the full-scale RCT (32). The study was approved by the National Research Ethics Service of the UK's National Health Service (ref 14/NW/0163) and registered with a clinical trial data base (ISRCTN reference: ISRCTN74643496).

Patients referred to CR programs in the UK are routinely sent a National Audit of Cardiac Rehabilitation assessment pack (9), which includes the Hospital Anxiety and Depression Scale (HADS) (33). Participants were recruited from three NHS cardiac rehabilitation services in the North West of England (University Hospital of South Manchester NHS Foundation Trust, Central Manchester University Hospitals NHS Foundation Trust, and East Cheshire NHS Trust). At the initial assessment appointment, CR staff reviewed each patient's medical notes for inclusion and exclusion criteria, initially screening for those who scored eight or above on either the HADS anxiety and or depression subscale, indicative of at least “mild” symptomatology. In addition, participants had to meet the Department of Health and/or British Association for Cardiac Prevention and Rehabilitation CR eligibility criteria, be aged 18 years or older, and have a competent level of English language (able to read, understand, and complete questionnaires in English). Patients who were eligible for the trial were invited to take part. Inclusion and exclusion criteria are provided in further detail in Wells et al. (32). Eligible participants were contacted by research assistants and participants were provided with further information on the trial and had the opportunity to ask questions about participation in the study. Participants were consented face-to-face with a research assistant either at the patient's home or at the site of their CR. Research assistants taking consent, the chief investigator and trial statisticians were blind to each patient's treatment allocation. Data monitoring, quality, and handling were undertaken by the Manchester Clinical Trials unit and project oversight was by an independent trial steering committee. The trial has been reported in line with CONSORT statement for the reporting of pilot and feasibility randomized controlled trials (34).

### Patient and Public Involvement

Patient and public involvement (PPI) has been extensively involved throughout the course of the study. PPI service users were first involved in grant development and commissioning of the research project. After which, PPI service users were subsequently involved in participant recruitment and ensuring that the number and length of outcome measures selected did not overburden participants, as well as aiding in recruitment and retention of participants. Specifically, PPI service users aided in co-producing a newsletter sent out to patients following 4-month follow-up to remind patients of upcoming follow-up questionnaires and updating participants about the study. PPI service users have also aided in co-producing a dissemination plan and aiding in dissemination of research findings.

### Participants

**Figure 1:**
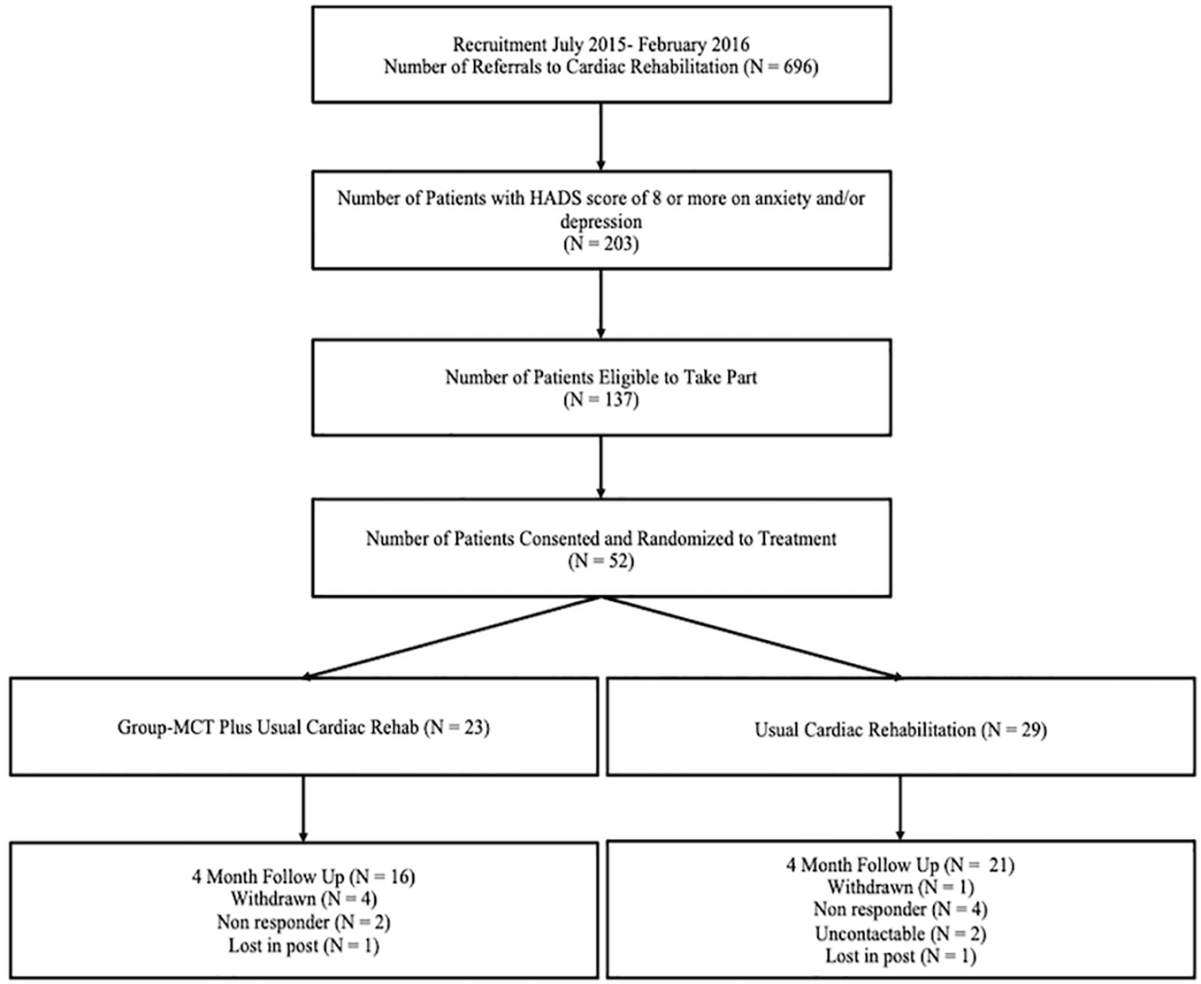
CONSORT flow diagram of patient recruitment.

The Consort diagram indicating patient flow is depicted in **Figure 1**. Fifty-two eligible patients were randomized to either usual cardiac rehabilitation (control) or to usual cardiac rehabilitation plus group metacognitive therapy (intervention).

### Randomization and Sample Size

Following informed consent, patients were randomly allocated to a trial condition in a 1:1 ratio using a minimization algorithm that incorporated a random component, in order to maximize balance between the two arms on sex distribution, HADS anxiety and depression scores, and hospital site. **Table 1** provides a summary of randomization variables by group. Randomization was conducted *via* a telephone link to the Manchester University Clinical Trial Unit (Manchester CTU).

**Table 1:** Sample characteristics by Randomization Variable per Group.

Arm	HADS Anxiety ≥ 8		HADS Depression ≥ 8		HADS Anxiety and Depression ≥ 8	
	Male	Female	Male	Female	Male	Female
TAU	6	3	2	2	9	7
TAU + Group-MCT	5	2	2	0	8	6

TAU, treatment as usual; MCT, metacognitive therapy; HADS, Hospital Anxiety and Depression Scale.

The target sample size was originally 50 patients (25 per arm), determined as sufficient to evaluate recruitment and retention rates for a full-scale trial as well as rates of completion of the intervention. This sample is also adequate for estimation of variability in outcome measures for which samples of 40 are considered sufficient (35). However, as a consequence of parallel recruitment across sites, 52 patients had consented by the end of the recruitment period and were included in the sample. Patterns of participant sex and HADS subscale scores resulted in 23 of these being allocated by computer to the intervention group and 29 to the controls. Sample characteristics are in **Table 2**.

**Table 2:** Baseline Demographic and Clinical Characteristics.

Demographic factors	Entire sample (N = 52)
	n (%)
Sex	
Male	33 (63.5%)
Female	19 (36.5%)
Ethnicity	
Any white	43 (82.7%)
All other categories	8 (15.4%)
Psychological therapies for anxiety or depression	
In the past	18 (34.6%)
Never	34 (65.4%)
Age	58 (9.6)
Employment:	
Economically active	18 (34.6%)
Unemployed	8 (15.4%)
Retired	16 (30.8%)
All other	10 (19.2%)
Educational qualification	
None	16 (30.8%)
School/vocational	22 (42.3%)
Diploma/degree	14 (26.9%)
Civil status:	
In relationship	26 (50.0%)
Separated	18 (34.6%)
Single	8 (15.4%)
Smoking status	
Never smoked	16 (30.8%)
Ex-smoker	31 (59.6%)
Current smoker	5 (9.6%)
Alcohol units per month	
None	21 (40.4%)
1 to 19	17 (32.7%)
20 to 49	7 (13.5%)
50 or more	7 (13.5%)
Age at first cardiovascular event	
Under 45 years	5 (9.6%)
45 to 54 years	21 (40.4%)
55 years and older	25 (48.1%)
Number of cardiac events	
None	38 (73.1%)
1	7 (13.5%)
2 or more	7 (13.5%)
BMI	
Underweight/normal	14 (26.9%)
Overweight	21 (40.4%)
Obese	15 (28.9%)
Number of comorbidities [mean (SD)]	5.1 (2.1)
**Outcomes**	**Mean (SD**)
HADS total	17.5 (5.7)
HADS anxiety	10.1 (3.6)
HADS depression	7.4 (3.5)
Impact of Event Scale-Revised (IESR)	32.6 (19.2)
Metacognitions Questionnaire 30 (MCQ-30)	62.3 (15.1)
EQ-5D-5L Utility	0.60 (0.3)
EQ-5D VAS	61.5 (18.5)
CAS-1R	394.1 (185.2)

### Treatment

#### Treatment as Usual (TAU; Control)

Treatment as usual was the CR program delivered at each study site in hospital and community settings. CR programs are comprised of an exercise component and an educational component. Both components are provided in group sessions over 8 to 10 weeks, of 45 to 60 min. The therapist-to-patient ratio in TAU exercise sessions is 1:5 for low- and moderate-risk patients and 1:3 for high-risk patients. In addition to exercise classes, educational seminars are delivered that focus on a variety of topics including lifestyle and medical risk factor management. TAU educational sessions varied by site and the extent of psychological components also varied. While all sites delivered sessions on relaxation which focused on breathing techniques and progressive muscle relaxation, the content of sessions on stress management differed. For example, two sites incorporated cognitive therapy methods (i.e. challenging negative thoughts, worry decision tree), while one site delivered psychoeducational information on stress. In addition, one site offered a 4-week stress management course as part of CR which included generating and sharing a case formulation based on Greenberger and Padesky (36), mindfulness techniques, and individual counseling with an occupational therapist.

#### Group-MCT Plus Treatment as Usual (Group-MCT; Intervention)

Group-MCT was delivered in addition to usual CR. Group-MCT was most often delivered on a different day to participants' CR sessions, and only four intervention patients attended CR and Group-MCT on the same day. Group-MCT was delivered in six sessions of 60- to 90-min duration and followed a treatment manual (37). Sessions focused on deriving a case formulation and socialization, practicing the Spatial Attentional Control Exercise (SpACE), detached mindfulness (DM), worry and rumination postponement, challenging metacognitive beliefs concerning the uncontrollability and danger of worrying and rumination, and developing a “helpful behaviors” plan. The manual contains techniques that help patients to change the type of relationship that they have with their negative thoughts, allowing them to step back and not engage in worry and/or rumination. One of the techniques, SpACE, is a brief auditory exercise consisting of instructions to allocate attention in different ways to discover control over thinking while inhibiting responses to spontaneous negative thoughts and prolonged attention capture by distractions. The technique is designed to increase mental flexibility so that negative thoughts and memories can be de-coupled from repetitive negative thinking. DM is a technique used in Group-MCT in which the patient learns to relate to thoughts and disengage from unhelpful coping strategies. Patients were asked to practice SpACE twice a day for homework and to practice applying DM to negative thoughts. SpACE practice was recorded in a personal diary and homework was reviewed at the start of each session with discussions around successes and difficulties experienced. However, data on practice were captured for analysis purposes.

### Therapists

Group-MCT was delivered by CR staff who had received a basic training in implementing the treatment manual. There were seven therapists, two therapists per site except one site where three therapists were trained. Therapists included occupational therapists, physical therapists, and CR nurses. Therapists completed a 2-day workshop delivered by the developer of MCT (AW). Training included didactic teaching, role play, discussion, and studying the treatment manual. In addition, therapists delivered the intervention to a pilot group of volunteers along with an additional 1-day workshop, which focused on enhancing the initial skills. Therapists received ongoing supervision on an occasional basis, while delivering the intervention.

### Measures

#### Outcome Measures

##### Hospital Anxiety and Depression Scale (HADS)

The Hospital Anxiety and Depression Scale [HADS; Zigmond and Snaith (33)] was specified as the primary outcome for the pilot trial and subsequent main RCT and is a 14-item measure that assesses symptoms of anxiety and depression, where items are scored on a 4-point Likert scale (0 to 3). The scale generates two subscale scores (anxiety and depression) and a total score. The total score was the pre-specified primary outcome measure. The HADS total score has demonstrated good internal consistency with Cronbach's alpha for the total score in cardiac patients ranging from 0.85 to 0.89 (38, 39).

##### Impact of Events Scale-Revised (IES-R)

The Impact of Event Scale-Revised [IES-R; Weiss and Marmar (40)] is a 22-item scale assessing symptoms of post-traumatic stress disorder across three subscales, intrusions, avoidance, and hyperarousal, plus a single total score. Each item is rated from 0 (not at all) to 4 (extremely) based on how distressing the item has been in the past week with respect to their cardiac event. The scale has shown high internal consistency across all three subscales: intrusion Cronbach's alpha = 0.87 to 0.94; avoidance Cronbach's alpha = 9.84 to 0.87; hyperarousal Cronbach's alpha = 0.79 to 0.91 (31–33); total Cronbach's alpha = 0.95 (41). The subscales also demonstrate moderate to high inter-correlations (rs = 0.52–0.87) (41, 42). We pre-specified the total score as the outcome for this study.

##### Metacognitions Questionnaire-30 (MCQ-30)

The Metacognition Questionnaire-30 [MCQ-30; Wells and Cartwright-Hatton (43)] is a 30 item measure that assesses metacognitive beliefs across five subscales: (1) positive metacognitive beliefs, (2) negative metacognitive beliefs regarding uncontrollability and danger, (3) cognitive confidence, (4) cognitive self-consciousness, and (5) need to control thoughts. Items are scored on a four-point Likert scale (do not agree to agree very much), whereby higher scores indicate greater maladaptive metacognitive beliefs. A total score is also derived and was used as the outcome measure for this study. The MCQ-30 possesses good internal consistency (Cronbach's alphas 0.72 to 0.93 for individual subscales) (43, 44).

##### Cognitive Attentional Syndrome-1 Revised (CAS-1R)

The Cognitive Attentional Syndrome-1 Revised [CAS1-R; Wells (45)] is a 10-item measure that assesses different aspects of the CAS across three subscales: coping strategies, positive metacognitive beliefs, and negative metacognitive beliefs. The measure was modified from the original CAS-1 (16) for use in PATHWAY. Items are rated on an 11-point Likert scale ranging from 0 (none of the time/not at all true) to 100 (all of the time/completely certain this is true), whereby higher scores indicate greater use of maladaptive coping strategies or maladaptive metacognitive beliefs. The psychometric properties of the CAS-1R have been evaluated in cardiac patients (46). The measure demonstrates acceptable internal consistency for two subscales: coping strategies (Cronbach's alpha = 0.88) and negative metacognitive beliefs (Cronbach's alpha = 0.65); however, internal consistency was low for the positive metacognitive beliefs subscale (Cronbach's alpha = 0.58). The scale demonstrates good construct validity and is a significant predictor of anxiety and depression in cardiac patients.

##### EQ-5D-5L

The EQ-5D-5L [EuroQol (47)] describes and values health across five dimensions: mobility, self-care, usual activities, pain/discomfort, and anxiety/depression (48). Response items range from no problem to extreme problem. The EQ-5D-5L can be calculated as a total score and also converted to a utility score. In addition, participants rate their overall health using a visual analog scale (VAS), which ranges from 0 (worst health imaginable) to 100 (best health imaginable).

### Statistical Analysis

Statistical analysis was principally descriptive. We assessed the acceptability of adding Group-MCT to usual CR in terms of rates of recruitment into the study (number agreeing to participate out of those approached, and number recruited per month), withdrawal or drop-out by the primary endpoint of 4 months (attrition rate), and numbers of MCT and CR sessions attended.

The feasibility of conducting a full trial was assessed with regard to completion of follow-up questionnaires (proportions of missing values, both overall and within trial arms), ability of the outcome measures to discriminate between patients (range of scores; floor or ceiling effects), and re-estimation of the required sample size based on the findings of this study (number of recruited patients required to detect an effect size of 0.4 on HADS total score at 80% power, controlling for baseline scores and allowing for attrition and clustering of patients within therapy groups). We examined therapist level of adherence to the treatment protocol since these individuals were non-mental health specialists without prior experience of delivering psychological treatments. An adherence checklist was used for this purpose, whereby therapists indicated the aspects of the protocol implemented in each session, if they had been missed and if so why. For example, in the first session adherence items included completing the case formulation, socializing patients to the model, practicing SpACE, and assigning homework. Consistently evaluated elements in each session included reviewing and assigning homework and practicing SpACE. A total adherence score was created for each session by summing the total number of elements completed in session.

## Results

### Participants

52 participants (19 female, 33 male) took part in the study. Patients had a mean age of 58.10 (SD = 9.61, range: 38–79). Participants ethnic origin was primarily white (82.7%); however, eight participants identified as the following ethnic origins: Asian Bangladeshi (n = 1), Asian Indian (n = 1), Asian Pakistani (n = 1), Any other Asian background (n = 1), Black Caribbean (n = 3), and any other Black background (n = 1). Patients had a range of heart conditions including: acute coronary syndrome (n = 31), revascularization (n = 26), heart failure (n = 9), angina (n = 3), implantation of cardioverter defibrillator (n = 1), heart valve repair/replacement (n = 8), and adult congenital heart disease (n = 2). At initial assessment, the mean anxiety score was 10.02 (SD = 3.27), and the mean depression score was 8.48 (SD = 3.13). 30 patients met criteria for both anxiety and depression, 16 patients met criteria for anxiety only, and 6 met criteria for depression only.

### Acceptability

Patients and therapists were asked about the acceptability of the treatment in a separate qualitative study that will be reported in detail elsewhere. The qualitative reports suggested that both patients and therapists found the treatment to be logical and appropriate for the problems being addressed.

### Feasibility Assessment

#### Recruitment

Participant recruitment took place over 8 months across three CR services in the North West of England. During this time 696 patients were referred to CR programs, of which 203 (29.2%) had a score of 8 or more on either or both HADS subscales. 137 patients met the study eligibility criteria, and 119 of these were provided with further information on the study. Of the 119, 14 started CR before they could be offered the trial (making them ineligible), 21 were missed (i.e. not screened for eligibility), while another 32 declined to participate. The remaining 52 (38% of those eligible to take part) were consented and randomized into the study. As such, over 8 months the rate of recruitment was approximately 6.5 patients consented and randomized per month.

#### Outcome Measures

**Figure 2:**
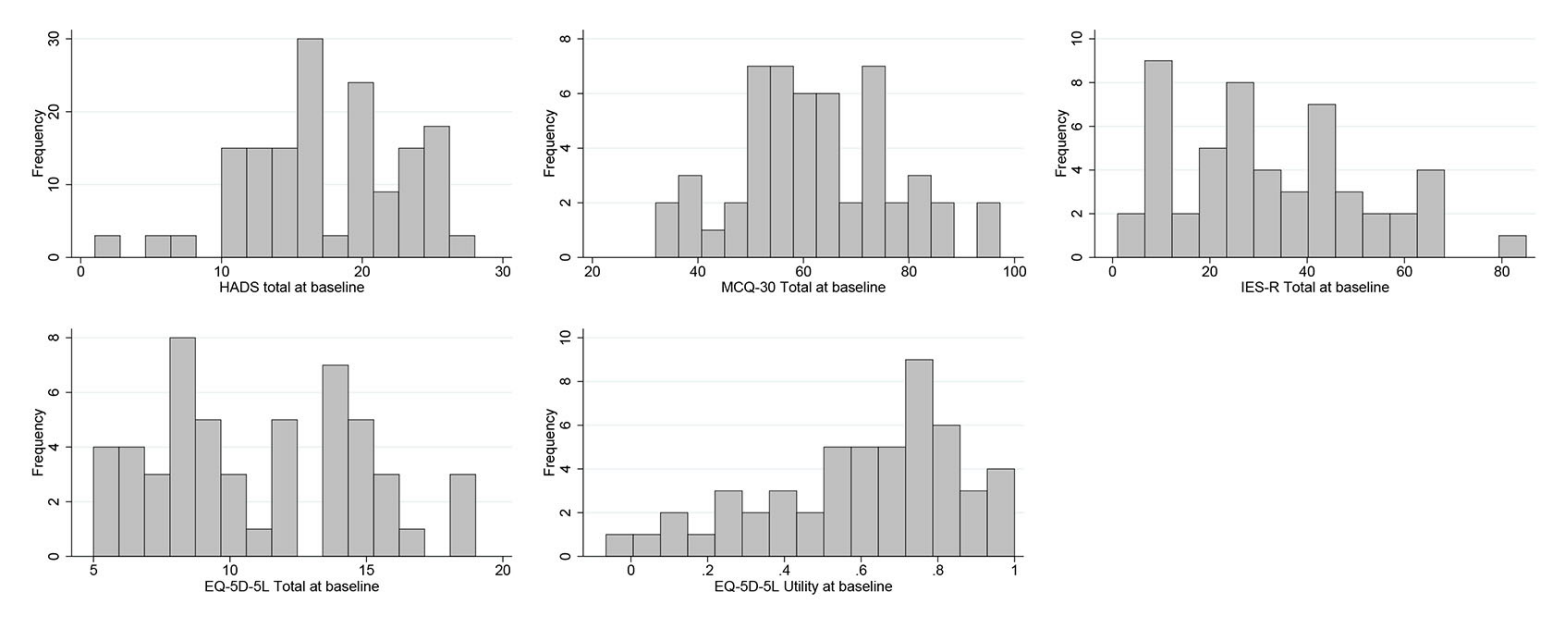
Outcome measure histograms.

To maintain the masking of the trial statistician (necessary for the inclusion of this data in the definitive RCT) the analyses of the outcome measures in the current study has been conducted on the full sample only. There was no missing data on any outcome measure at baseline. **Table 3** provides a summary of the descriptive statistics for each outcome measure and **Figure 2** provides a histogram for each. All questionnaires demonstrated a good range of observed scores, covering the majority of the possible score range, and with little in the way of floor or ceiling effects. We note that HADS scores at baseline could (and did) go below the minimum of eight that applied at the time eligibility was assessed.

**Table 3:** Descriptive Statistics on Outcome Measures at Baseline.

Outcome measure	Sample Size	% Missing	Median (inter-quartile range)	Minimum and maximum observed scores	Minimum possible score (% scoring minimum)	Maximum possible score (% scoring maximum)
Hospital Anxiety and Depression Scale (total score)	52	0	17 (13.5–22)	1, 28	0 (0%)	42 (0%)
Impact of Event Scale-Revised (total)	52	0	30.5 (18–45)	1, 85	0 (0%)	88 (0%)
Metacognitions Questionnaire 30 (total)	52	0	60 (52.5–72.6)	32, 97	30 (0%)	120 (0%)
EQ-5D-5L (total)	52	0	10 (8–14)	5, 19	5 (7.7)	25 (0%)
EQ-5D-5L utility score	52	0	0.66 (0.45–0.80)	−0.065, 1	−0.594 (0%)	1 (7.7%)

#### Treatment Attendance and Retention

##### Usual Care Condition

CR exercise sessions were well attended, with a median of six sessions attended and 58.6% of patients attending at least six sessions.

##### Intervention Condition

**Figure 3:**
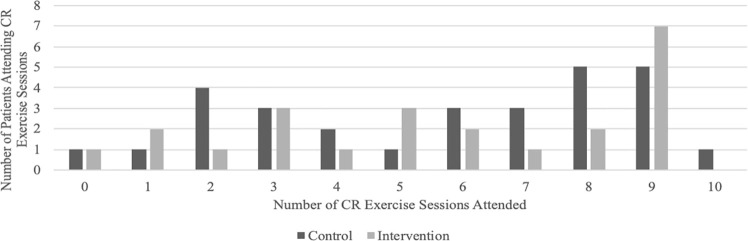
Number of cardiac rehabilitation exercise sessions attended by group.

Patients in Group-MCT had a high attendance rate at CR exercise sessions, with a median of six sessions attended and 52.2% of patients attending at least six sessions. **Figure 3** summarizes the number of CR exercise sessions attended by group.

Overall, attendance at Group-MCT was high, with 13 patients (56.5%) attending at least four out of six sessions of which 11 (47.8%) attended all six. However, five patients (21.7%) did not attend any sessions and five (21.7%) attended only one or two.

#### Four-Month Follow-Up

At 4-month follow-up 72.4% and 69.6% of control and intervention group participants, respectively, returned follow-up questionnaires.

One control group participant formally withdrew from the study (3.5%), though this was immediately after randomization. Six control group patients did not return questionnaires at follow-up (20.7%), and one questionnaire was lost in the post (3.5%).

In comparison, four (17.4%) MCT-arm participants formally withdrew from the study before 4-month follow-up. Reasons for withdrawal included no longer having enough time to commit to the study (n = 1), and no longer being interested in the study (n = 3). However, as only two additional MCT-arm patients failed to return the 4-month follow-up the overall response was similar. One questionnaire was lost in the post (4.4%).

#### Group-MCT Adherence

We examined therapists' level of adherence to the treatment protocol since the treatment was to be administered by non-mental health specialists with no experience of delivering psychological treatments. Therapists completed an adherence checklist at the end of each session to indicate which aspects of the session had been completed. Each site had two CR staff members trained to deliver MCT, one site had three staff members trained. Adherence to the protocol was high across all three sites, with an average adherence rating across sites of 98.2%. Each site deviated from the protocol once; one site was unable to practice SpACE in session three as the session over-ran, while the remaining two sites both failed to review the previous session's homework. One site failed to review the homework in session five while the other site did not review the homework in session six.

#### Eligibility Rates Before Baseline

There was an unavoidable delay between patients having their initial CR assessment appointment and their subsequent study baseline assessment. This period was on average 7.5 days (SD = 5.95), but went as high as 31 days; however, baseline assessment was always completed prior to a patient's first cardiac rehabilitation session (i.e. first exercise class). HADS scores may change over this time, resulting in patients falling below the eligibility criteria of 8 points on the anxiety or depression subscale, and some may even spontaneously experience full clinical recovery. To assess the extent of these risks, we examined the change in HADS scores between initial assessment and study baseline. Five patients (10%) made a clinically significant improvement (i.e. made a seven-point change on the HADS, calculated using (49), and two fell below the cutoff score of 8 points. One patient was classified as recovered (i.e. made a seven point change on the HADS and crossed the cutoff score).

#### Sample Size Confirmation

The Pathway RCT is a superiority trial comprising of an initial internal pilot followed by a main trial. The main trial was designed to detect an effect size of 0.4 on HADS total and to have 80% power not including the pilot sample. Under assumptions of 25% attrition, a correlation of 0.5 between baseline and follow-up outcome scores, mean therapy group size of 5.75, and intra-cluster correlation coefficient (ICC) of 0.05, we estimated that a total recruitment sample of 230 patients was required for the main trial. Should merging with the internal pilot data prove feasible the final combined sample would be 280, and the power 85%.

We revised this estimate on the basis of the results of this internal pilot, which indicated an attrition rate of 35%, over-time correlation of 0.5 (unchanged), mean group size of 3 and ICC of 0.05 (assumed). Since no substantial changes were made to the trial procedures or instruments following the pilot, with the consent of our steering committee and funders the decision was taken to merge the pilot data with the main trial. Considering the available time and resources, it was also decided to increase the recruitment target to 332, to give the full study 90% power to detect the desired 0.4 effect size.

#### Adverse Events

Adverse events and serious adverse events were monitored for individuals in the intervention group. No adverse events were reported. For further details on the safety reporting protocol for the trial see (32).

## Discussion

Anxiety and depression are common in cardiac patients; however, current psychological interventions for cardiac patients are limited in efficacy, and as such novel psychological interventions are needed. The current study evaluates the acceptability of Group-MCT in cardiac patients and the feasibility of conducting a full randomized trial to compare Group-MCT plus treatment as usual to treatment as usual in this patient population.

Group-MCT was found to be both an acceptable and feasible treatment to deliver to cardiac patients with anxiety and depression. Recruitment and retention of participants to a psychological trial is often difficult (50); however, the current study successfully recruited to target. Recruitment rates were in line with previous studies evaluating psychological interventions within CR patients, whereby approximately 35% of eligible patients agreed to take part in research (51). The HADS scores of participants in the study were also similar to those obtained in routine practice in the UK (52), which supports the generalizability of the study to clinical settings. Although the study did recruit to target there were challenges with recruitment, for example, some patients were not screened for eligibility due to limited CR staff or time to complete eligibility questionnaires. As such, future studies should consider providing additional support to CR staff during assessment clinics to assist with screening patients for eligibility. Reasons for not taking part in the trial included not having the time to commit to the study, returning to work, and not being interested in the study. Interestingly, one patient noted that despite scoring high on the HADS they did not feel as though they were anxious or depressed and therefore did not feel as though the research study was suitable for them.

Completion rates of CR were high at 75%, which is in line with The National Audit of Cardiac Rehabilitation Annual report, which highlights that nationally in the UK 77% of patients complete CR (9). Completion of CR did not differ by group or by site, suggesting that the addition of Group-MCT does not impact on patient willingness to attend CR. Attendance at Group-MCT was moderate to good, with more than half of all patients attending at least four out of six sessions of Group-MCT, and most of these attending all six. We consider attendance at a minimum of four sessions to represent sufficient exposure to benefit appreciatively from the intervention. However, around one fifth of intervention patients did not attend any sessions, with a majority of these not providing a reason for non-attendance. Attendance at Group-MCT in the current study was slightly lower than some previous studies, but comparisons are difficult to interpret because not all other studies report attendance rates, and the studies are not within a cardiac sample.

Retention of participants in both trial groups was high. There was some evidence for a higher—though still small—number of participants randomized to Group-MCT formally withdrawing from the study, but this was balanced by a greater rate of return of follow-up questionnaires from this group. There appeared to be no indication that attending Group-MCT led to a reduction in attendance at CR sessions. It is important to note that reasons for withdrawal from the study may have been influenced by the format of delivery of the intervention. Some patients may not feel comfortable attending a group intervention and may have preferred a psychological intervention delivered on a one-to-one basis.

The study had a high rate of return of follow-up questionnaires with over 70% returned. The study used a broad range of strategies to encourage returns including phone calls to check that patients had received their follow-up questionnaire packs, reminder phone calls asking patients to return them, and incentivizing return of questionnaires. In addition, we also provided patients with a range of options which included completing questionnaires over the phone and face-to-face with a research assistant. While a range of options were provided future studies should also consider offering electronic data collection, which may help to increase questionnaire return rates.

While the therapists reported a high level of adherence to the manual we were unable to assess the quality of therapy delivered throughout. This was due to limitations with audio recording therapy sessions. In future, trials should not only assess adherence to the manual but also quality of treatment delivery *via* an independent rater.

On the basis of the results reported here extension of the study to a full-scale trial was recommended. The results of the feasibility study were reported to the trial steering committee and the funder (NIHR), and the decision to extend recruitment to a full-scale RCT was supported.

## Conclusions

The implementation of psychological treatments for anxiety and depression in physical health pathways presents several challenges. Existing treatments have limited efficacy and are not widely accessible. Furthermore, physical health care providers typically lack the knowledge and experience necessary to deliver complex psychological interventions. With these barriers in mind, we evaluated the feasibility and acceptability of running a trial of MCT in cardiac rehabilitation patients and delivered by non-mental health specialist staff. Our feasibility study was implemented as an internal pilot study, with the intention that if findings were favorable the study would be extended by continuing recruitment to a full-scale RCT.

The results suggest that a trial of MCT within the rehabilitation pathway is feasible. The addition of MCT to rehabilitation did not appear to have a negative impact on CR retention. Most participants completed our a-priori definition of a minimally effective dose of the intervention. Completion rates of measures were good suggesting they did not over-burden participants. The treatment approach also appeared feasible and acceptable to health providers as we observed high levels of therapist adherence to the protocol.

## Data Availability Statement

The datasets generated for this study are available on request to the corresponding author.

## Ethics Statement

The studies involving human participants were reviewed and approved by National Research Ethics Service of the UK's National Health Service (ref 14/NW/0163). The patients/participants provided their written informed consent to participate in this study.

## Author Contributions

AW, DR, PF, LD, AH, and PD were involved in study conception or design, and contributed to the first and subsequent drafts of the manuscript. DR and CH contributed to data analysis. LC drafted the manuscript and contributed to data analysis. DR, CH, AW, and LC contributed to interpretation of results. All agree to be accountable for all aspects of the work ensuring integrity and accuracy.

## Funding

This study represents independent research funded by the National Institute for Health Research (NIHR) under its Programme Grants for Applied Research (PGfAR) Programme (Grant Reference Number RP-PG-1211-20011). The views expressed are those of the author(s) and not necessarily those of the NIHR or the Department of Health.

## Conflict of Interest

AW is the developer of metacognitive therapy and a co-director of the Metacognitive Therapy Institute.

The remaining authors declare that the research was conducted in the absence of any commercial or financial relationships that could be construed as a potential conflict of interest.
